# EBOLA VIRUS DISEASE PREPAREDNESS IN COUNTRIES BORDERING DEMOCRATIC REPUBLIC OF CONGO: LESSONS FROM WEST AFRICAN OUTBREAK

**DOI:** 10.21010/ajid.v14i2.6

**Published:** 2020-07-31

**Authors:** Lawrence Okoror, Otridah Kapona, Mpanga Kasonde, Tobin Alice E, Asogun Danny, Otukoya Ashiat M

**Affiliations:** 1 Department of Microbiology, Federal University, Oye-Ekiti, Nigeria; 2 Zambia National Public Health Institute, Kabulonga, Lusaka, Zambia; 3 Zambia National Public Health Institute, Kabulonga, Lusaka, Zambia; 4 Institute of Lassa Virus Research, Irrua Specialist Teaching Hospital, Irrua, Nigeria; 5 Institute of Lassa Virus Research, Irrua Specialist Teaching Hospital, Irrua, Nigeria; Member PANDORA consortium; 6 Department of Microbiology, Federal University, Oye-Ekiti, Nigeria

**Keywords:** Ebola, EVD, Preparedness, Response, Infection Prevention and Control

## Abstract

**Background:**

Ebola virus disease ravaged three West African countries in the wake of 2014 which was seen as the deadliest Ebola Virus Disease (EVD) outbreak in history. Several lessons were taken out of the West African outbreak one of which is the lack of preparedness by countries in the region.

**Materials and Methods:**

This paper looked at the mistakes of the West African outbreak and reports how such mistakes were corrected in the current outbreak going on in the Democratic Republic of Congo (DRC). Preparedness efforts are currently taking place in countries bordering DRC which included quick detection and response to an eventual EVD event.

**Results:**

This paid off on several occasions when cases from DRC to Uganda were quickly detected and response was as quick as possible. Preparedness carried out in Countries bordering DRC included setting up of Rapid Response Team (RRT) and training of these teams both at country and regional level. All members of the RRT were trained in all areas of readiness which included community engagement, laboratory, logistics, surveillance, case management, sample collection, packaging and shipment as well as Infection Prevention and Control (IPC).

**Conclusion:**

These trainings have led to readiness to an eventual EVD event. Countries now have the ability to respond quickly with better Emergency Operation Centre (EOC) for EVD.

## Introduction

Ebola Virus Disease (EVD) is a severe and often fatal disease in humans (WHO 2017). The disease ravaged three countries in West Africa in 2014 which included Guinea, Liberia and Sierra Leone. The case fatality ranges from 50% to 90% in clinically-diagnosed cases (Hewlett and Hewlett 2005). The West African outbreak sees Sierra Leone accounting for the highest number of cases with about 50% of the 28160 cases across the three affected countries (WHO 2017). Nigeria also witnessed the outbreak when an infected individual traveled from Liberia to Nigeria. Prevention still remains the only option at curtailing the disease (WHO 2017).

The first case of EVD was first reported in Zaire (Democratic Republic of Congo) and the virus was named Ebola virus following its discovery near River Ebola (Le point, 1976 and Le mond 1976).

Ebola virus belongs to the family Filoviridae which consists of a group of thread like viruses with sand-like particles. They belong to the order Mononegavirales. The virus is a U shaped pleomorphic particle which could also be a “6” or circular shaped viruses. The virion most frequently seen under the electron microscope is generally long tubular structure. The virion has a genome which contains one molecule of linear or helical, single stranded, negatively sensed RNA (Netesove *et al.*, 2000). EVD could be caused by any of the five strains of Ebola virus. The most common of the strains is the Zaire Ebola Virus (Netesove *et al.*, 2000 and Pringle, 1998), the other ones include Sudan Ebola Virus which was introduced as a new strain in 1978; WHO describing the 1976 outbreak of Ebola in Sudan as being caused by the Sudan Ebola Virus strain (Geisebert and Jahrling 1990). The Tai forest Ebola virus strain was responsible for the Ivorian Ebola Virus outbreak and was recognized as a new strain of Ebola virus in 1995. However, in 2002 it received a new name and was now referred to as Cote d’voire Ebola virus strain (Geisebert and Jahrling 1990). Bundibugyo Ebola virus strain was first discovered in Bundibugyo district of Uganda. The Reston Ebola Virus strain was recognized as a new strain of Ebola virus in 1990 (Kuhn *et al.*, 2010; Morvan *et al.*, 1999).

Ebola virus was first introduced into humans by those who made contact with a dead infected animal carcass or infected animal hunted down, which is the first source of the virus and this was followed up by human-to-human infection (Geisbert and Jahrling 2005). Transmission normally occurs when people come in contact with tissues or bodily fluid of infected animals or fomites of infected individuals (Hewlett and Hewlett 2005). Most outbreaks have been caused by human-to-human transmission after the index case as reported by Leroy *et al.*, (2005). Although the main vector of Ebola virus is still unclear, the most likely vector is the fruit eating bat, specifically *Hypsignathus monstrosus* (the hammer-headed fruit bat), *Epomops franqueti* (Franquet’s epaulets fruit bat), and *Myonycteris torquata* (the little-collared bat (Leroy *et al.*, 2005). Although other animals have been suspected to transmit the virus, it is also suspected that these animals may have made contact with materials contaminated by fruit bat (Kangoy et al. 2016). There have since been no studies that have confirmed rodents as reservoir of ebola virus. In 2001 and 2004, traces of ebola virus were found in Chimpanzees and gorillas which eventually became sources of human infection (Kangoy *et al.*, 2016). These animals, though sources of human infection in that outbreak, could not be seen as a reservoir because of the very high rate of deaths among the animals (Kangoy *et al.*, 2016). Once contracted by humans through contact with infected animals, the disease is easily spread within the human population through human-to-human transmission. The clinical features of Ebola Virus include a first phase of influenza-like illness with an onset of non-specific illness characterized by symptoms such as high fever, headache, arthralgia, myalgia, sore throat, and malaise with nausea (Hewlett and Hewlett 2005). The second phase of the infection involves persistent fever not responding to antimalarial treatment or antibiotics, headache, intense fatigue which is followed by diarrhea, abdominal pain, and anorexia and vomiting. The 3^rd^ phase involves a pseudo-remission which is around day 7-8 of the infection, and the patient feels better, the health situation improves at this phase and some patients may recover at this phase. Day 9 may present with aggravation of the disease, symptoms like respiratory disorder sets in, which includes dyspnea, throat and chest pain, cough, hiccups, hemorrhagic diathesis, bloody diarrhea, hematemesis, conjunctival injection, gingival bleeding, nose bleeds and bleeding at the site of injection consistent with disseminated intravascular coagulation, skin manifestations (petechiae, purpura, morbilliform skin rash), neuropsychiatric manifestations (prostration, delirium, confusion, coma), and cardio-vascular distress and hypovolemic shock (death), (Kangoy *et al.*, 2016).

It should be noted that patients do not transmit ebola during the incubation period but only becomes infectious once they start showing symptoms (Hewlett and Hewlett 2005). One of the major problems in lack of early detection of EVD is that they mimic other tropical diseases like malaria and typhoid fever which make early detection difficult and patients may get to the aggravation phase before the disease is finally confirmed through laboratory testing (WHO 2017).

As of today there have not been any drug licensed for EVD and measures to preventive measures have to be taken including avoiding carcasses of animals of unknown death, isolation of individuals suspected of EVD. The West African Outbreak was heralded by several complications in outbreak management, especially at the beginning of the outbreak. Such complications encourage the spread of the disease across borders of which lessons were put into practice with the current DRC EVD outbreak which started in 2017.

## Materials and Methods

Information was obtained from documentation and diary of personal events during the participation in the outbreak response in Sierra Leone and papers published online and prints were accessed. Those documentations and publications were compared to the preparedness efforts carried out in Zambia and other Countries bordering the DRC. Such documentations included Response activities, preparedness, Infection Prevention and Control, Epidemiological surveillance, Social engagement and risk communication, Laboratory and psychosocial support. Every section of the preparedness in countries bordering the DRC were compared to that of West Africa to see if there were improvement in preparedness and response in these countries as compared to Sierra Leone and West Africa in general. Quantitative analysis of all the training were also investigated as there were little or no training specific to EVD before the West African outbreak. Effectiveness of the Rapid Response Teams set up by countries was determined through the type on trainings conducted for the team and also percentage of different areas of HCW that were involved in RRT.

### Response

Early response in Sierra Leone, for example, was poor as there was no cooperation from the communities due to lack of preparedness. There was no in-country ready team for outbreak of such magnitude, hence, the health system of the country was completely overwhelmed. Even early outbreak warning was ignored. Countries were not ready for outbreak of such magnitude. Early detection and response to an outbreak is the key to successful management of the outbreak. In Democratic republic of Congo, there was early detection which led to early response, a carryover of lessons learned from the West African outbreak. There was sufficient International assistance starting from the beginning of the outbreak

However, all these efforts yielded only limited results due to insecurity in DRC. Lesson learned from West African outbreak, is the need for quick intervention.

### Preparedness

**Fig 1:**
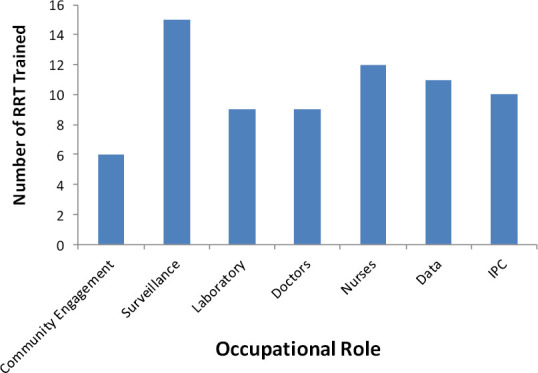
RRT members trained to combat EVD in ZAMBIA

**Fig 2:**
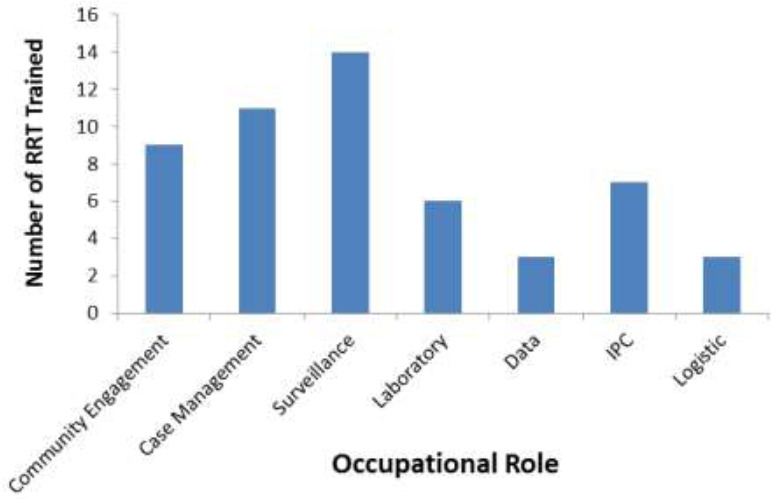
EVD RRT members trained in ZAMBIA by WHO African Regional Office

In the West African outbreak, it was obvious that preparedness was lacking, as a lesson from West Africa, neighboring countries to DRC engaged in EVD preparedness. In the West African outbreak, health care workers were unaware of the disease they were dealing with due to lack of preparedness and training which led to massive deaths of healthcare workers (Kagoy *et al.*, 2016 and Leroy *et al.*, 2005). As part of preparedness, the countries around DRC established functional Rapid Response Teams. Preparedness in DRC neighboring countries were categorized according to risk assessment and preparedness activities were conducted based on this categorization. There were priority 1 and 2 countries. For example, South Sudan and Uganda were in the priority 1 countries, while Zambia which has a long border with DRC, was in priority 2. In order to forestall what happened in the West African outbreak, EVD preparedness was initiated by the WHO in the countries sharing borders with DRC, most of these countries share porous borders with DRC and open water ways which boosted trade among the countries. Firstly, the Rapid Response Team (RRT) was set-up to handle any form of outbreak in these countries, with special attention to EVD; the Emergency Operation Centre (EOC) was enhanced and strengthened. Training of the RRT in outbreak management was done; it was to make sure that all the members of RRT were familiar with all areas of EVD response. These trainings were sponsored by WHO in both the Country offices and the African Regional Office. These trainings by WHO AFRO took place in all priority 1 countries which included South Sudan and Uganda. Apart from these general trainings, there were also specific trainings of different areas involved in the RRT which included IPC, sample collection, laboratory, surveillance, reporting and risk communication. The IPC teams were again trained differently; the surveillance teams were also trained as well as the laboratory team specific to their duties which is more detailed than what was done during the RRT training. A total of 66 medical personnel were trained by the WHO country office in Zambia as RRT in different field (Fig. 1), while the WHO African Regional office trained another batch of 42 RRT in different professional fields (Fig. 2) and in different locations in order to, as much as possible, accommodate those who could not attend the first training.

### Infection Prevention and Control

Prior to the EVD outbreak in West Africa, nearly all the countries where the outbreak occurred only have basic, with no national IPC guidance, no dedicated staffs in government places and healthcare facilities to ensure proper implementation of IPC best practices (Pourrut *et al.*, 2005; Baker *et al.*, 2001). Hence, in order to prepare DRC neighboring countries for an eventual EVD, event IPC best practices were instituted, though Zambia have been battling different form of cholera outbreaks, yet the IPC practices were still poor. It was recommended that the existing IPC manual be reviewed and adapted to EVD, since none existed for EVD. Several strategies to contain an eventual EVD outbreak were instituted like the construction of a dedicated EVD treatment center and several isolation units. In Uganda, the IPC practices took place, which included implementation of IPC procedures to safe and dignified burials.

Reports have it that there was a positive association between health care structure, IPC performance, and the number of IPC trained staffs within healthcare facility IPC cascade within an health care facility, IPC cascade training by IPC-trained staff, compared to no cascade training, is more likely to result in higher IPC performance, regardless of the type of health care structure and whether the structure is certified or not (i.e. whether it has government permission to provide health care) (Savolainen-Kopra *et al.*, 2012; Cohen 1998; Mott *et al.*, 2007).

Infection Prevention and Control mechanisms in health care settings like donning of full Personal Protective Equipment (PPE) by health care workers before seeing a suspected case, full disinfection of areas where an infected case is suspected.

Reports have it that most healthcare workers that lost their lives in the West African outbreak (2014-2016) actually got infected during doffing of Personal Protective Equipment (PPE), hence during the preparedness training, emphasis were placed on donning and doffing of full PPE and also IPC procedures before and after seeing a patient, hand washing techniques, production of chlorinated water, local production of alcohol based hand scrub. Several decontamination “stopovers” after seeing a patient were introduced. Frontline health workers are now being vaccinated, high risk communities are also been vaccinated which included contacts and contact of contacts. This has led to reduction in transmission among health workers as well as break in chains of transmission. Uganda practiced proper point of Entry (PoE) IPC procedures during preparedness as well as proper transportation of patients and samples.

### Epidemiological Surveillance and Social Engagement and Risk Communication

The outbreak in West Africa was politicized with a section of the population refusing to cooperate with surveillance officers. There were also situations where cases were hidden by the community until death sets in, with engagement in secret burials leading spread of EVD in the community. There were not enough community engagement activities and not a proper risk communication. In Zambia for example, the RRT were trained on how to engage the community during surveillance activity, this was well emphasized by the AFRO WHO team during their training of the RRT in Zambia. Delay in laboratory results was also a problem faced by the epidemiological surveillance in Sierra Leone.

In order to solve this problem in the current preparedness effort, the laboratory was represented in the RRT to liaise between the laboratories and the team. The use of proper epidemiological forms was also emphasized during the training and the RRT were trained on how to use these forms and proper form of reporting format of any surveillance outcome. This will save undue delays in getting epidemiological reports noticed during the West African outbreak. Also the danger of improper administration of epidemiological forms have caused problems in the past as well as proper documentation of samples due to improper filing of all types of forms leading to misplacement of results. All these pose a great danger and limits all efforts towards breaking the transmission chain and also do compromise patient’s health.

Risk communication was completely absent in Sierra Leone prior to the EVD outbreak as the outbreak was unexpected and even at the earlier stage, the risk of contracting EVD was not appreciated. The population did not know to what extent the risk of EVD until the 3 day lock down. Community education is required to get the people well informed about the danger of such practices. This was corrected during the preparedness activities by extensive communication of the populace especially those areas bordering DRC as there is free movement between countries. Areas along Lake Tanganyika remain open to all forms of free fish trade and efforts were concentrated in these areas. This was adequately practiced in Uganda and South Sudan.

### Laboratory

In Sierra Leone, the laboratories were not prepared for an EVD outbreak and even for other reportable diseases. They were ill-prepared and this was a major problem during the outbreak which affected surveillance activities and the eventual spread of EVD. Eventually the different partners responded with different laboratories which included erection of new and efficient ones through to mobile laboratories which actually helped in speeding up activities in the field, but was not quick enough to stop the early spread of the disease. In Zambia, for example, the laboratories were not ready for any eventual crossover of EVD from neighboring DRC. In order to get the country prepared, the laboratories in countries along the DRC border were assessed with a view of up lifting them for better performance in the case of a spill-over from DRC.

Expert Ebola machines (Genxpert) were installed in strategic laboratories close to the border to DRC in Zambia for quick diagnoses and reporting of results; all these were lacking in West Africa at the beginning of the outbreak. Rapid diagnosis kits were also provided to those responsible in Zambia for onward distribution to these hard-to-reach areas for quick presumptive diagnosis following criteria to be developed by the regulatory authorities. During the West African outbreak, this was lacking and only introduced in the middle of the outbreak. Adequate training for laboratory personnel in sample handling and quick processing was done in Zambia as most of the laboratory workers were ill-equipped, this greatly affected surveillance activities in the West African outbreak. Specific training dedicated to the laboratory alone was conducted severally for all those laboratory workers situated very close to DRC border.

### Psychosocial Support

Fright factor was also a factor that was noticed in healthcare workers in West Africa, especially after the death of close relatives and colleagues. This led HCW to stay away from the response or develop nonchalant attitudes. In Zambia, however, this was taken-care of by continuous and re-training of healthcare workers in sample reception and sample handling, use of proper Personal Protective Equipment. Psychosocial support experts were also engaged to support HCW in the eventual EVD cross-over from DRC.

## Conclusion

Based on the lessons from the West African outbreak, some achievements have been recorded in Zambia, Uganda, South Sudan, Tanzania and so forth, although a lot still need to be done if Zambia is to achieve complete readiness in case of an EVD event. Though there are some positive responses in the preparation and readiness of the RRT due to regular trainings, Zambia needs to adequately formulate a strategy for surveillance activities should an EVD event occur. Risk communication also needs to be more effective to break any form of fright factor that might be experienced by the healthcare workers and the population in general. Reporting processes also need to be improved. Most importantly, the laboratories must be improved to cater for an EVD event, some efforts may have been put into training personnel but these efforts will not yield significant results if the laboratories cannot release reliable results considering timeliness for surveillance activities. Provision of adequate laboratory equipment and reagents are a key to nipping in the bud any EVD event that is likely to spill over from DRC. Adequate resources must be mobilized to achieve any significant results during preparedness. Although there is no EVD report from Zambia, Zambia must immediately begin active surveillance in order to quickly detect the likelihood of any hidden EVD case. This will ensure case-by-case follow up of contacts and eventual referrals, and also to determine the scope of the outbreak. Social mobilization must also be stepped up at this stage of the outbreak in DRC, and be put in readiness at all time. In Zambia, all the 6 suspected cases tested proved negative, these cases were detected early due to proper preparedness effort.

**List of Abbreviations:**
WHO: —World Health Organization
IPC: ---- Infection Prevention and Control
RRT: ----- Rapid Response Team
EVD: ------ Ebola Virus Disease
DRC: ----- Democratic Republic of Congo
EOC: ----Emergency Operation Centre
AFRO: ----- African Regional Office
PPE: -----Personal Protective Equipment
PoE: -------- Point of Entry.
